# Pseurotin D Inhibits the Activation of Human Lymphocytes

**DOI:** 10.3390/ijms22041938

**Published:** 2021-02-16

**Authors:** Daniela Rubanova, Petra Dadova, Ondrej Vasicek, Lukas Kubala

**Affiliations:** 1 Institute of Biophysics of the Czech Academy of Sciences, 612 65 Brno, Czech Republic; rubanova@ibp.cz (D.R.); dadova@ibp.cz (P.D.); 2 Department of Experimental Biology, Faculty of Science, Masaryk University, 625 00 Brno, Czech Republic; 3 International Clinical Research Center, St. Anne’s University Hospital, 656 91 Brno, Czech Republic

**Keywords:** pseurotin, lymphocyte, STAT3, STAT5, proliferation

## Abstract

Background: Pseurotins, a family of secondary metabolites of different fungi characterized by an unusual spirocyclic furanone-lactam core, are suggested to have different biological activities including the modulation of immune response. Purpose: Complex characterization of the effects of pseurotin D on human lymphocyte activation in order to understand the potential of pseurotin to modulate immune response in humans. Methods: CD4+ and CD8+ T cells and CD19+ B cells isolated from human blood were activated by various activators simultaneously with pseurotin D treatment. The effects of pseurotin were tested on the basis of changes in cell viability, apoptosis, activation of signal transducers and activators of transcription (STAT) signaling pathways, production of tumor necrosis factor (TNF)-α by T cells, expression of activation markers CD69 and CD25 on T cells and Human Leukocyte Antigen–DR isotype (HLA-DR) on B cells, and the differentiation markers CD20, CD27, CD38, and immunoglobulin (Ig) D on B cells. Results: Pseurotin D significantly inhibited the activation of both CD4+ and CD8+ human T cells complemented by the inhibition of TNF-α production without significant acute toxic effects. The Pseurotin D-mediated inhibition of T-cell activation was accompanied by the induction of the apoptosis of T cells. This corresponded with the inhibited phosphorylation of STAT3 and STAT5. In human B cells, pseurotin D did not significantly inhibit their activation; however, it affected their differentiation. Conclusions: Our results advance the current mechanistic understanding of the pseurotin-induced inhibition of lymphocytes and suggest pseurotins as new attractive chemotypes for future research in the context of immune-modulatory drugs.

## 1. Introduction

Small organic molecules isolated from fungi represent highly attractive compounds that have the potential for the modulation of specific physiological processes by affecting individual cellular pathways. A particularly interesting group of such fungal secondary metabolites is pseurotins, ergot-like alkaloids containing a 1-oxa-7-azaspiro[4.4]non-2-ene-4,6-dione core, that comprise a family with over twenty-five members such as azaspirene and synerazol [1,2,3,4]. Pseurotin A, the first described member, was isolated in 1976 from the fermentation broth of *Pseudeurotium ovalis* Stolk (Ascomycetes) by Bloch [5]. Later, pseurotins A–E were isolated by Tamm and coworkers [6]. Further, pseurotins are also produced by *Aspergillus fumigatus* (e.g., pseurotin A, 8- O-demethylpseurotin A, pseurotin D, F1/F2, 11- O-methylpseurotin A, and synerazol) as well as by *Neosartorya* sp. producing azaspirene and *Penicillium* strains producing pseurotin A [7].

Pseurotins have interesting biological activities. In addition to their antifungal and antibiotic activities [8], pseurotins were shown to modulate cell differentiation [9], to possess anti-angiogenic activity, to inhibit endothelial cell migration [10,11,12], and to regulate enzymes of cellular metabolism [13]. Importantly, interesting effects were observed on the function of the immune system. In our previous studies, we observed that the natural pseurotins A and D significantly inhibited the proliferation of murine macrophages, which was accompanied by downregulation of the expression of cyclins and mitochondrial respiration via the inhibition of particular signal transducers and activators of transcription (STAT) and mitogen-activated protein kinase (MAPK) signaling pathways [14]. Further, these two natural pseurotins (A and D) and a collection of fully synthetic pseurotin analogs were shown to inhibit immunoglobulin (Ig) E production and the proliferation of mouse B cells stimulated by interleukin (IL) 4 and *E. coli* lipopolysaccharide via the inhibition of phosphorylation of STAT proteins in stimulated B cells [15]. In general, these data showed that pseurotin D was more potent than the most studied pseurotin, pseurotin A [14,15]. Moreover, synerazol and 10-deoxypseurotin A showed IgE inhibitory activity and immunosuppressive activity inhibiting mixed lymphocyte reaction [16]. Thus, pseurotins can be viewed as natural products with the potential to specifically affect immune system functions.

Dysregulated control of the immune system has major health consequences and is behind the development of numerous diseases including various autoimmune and allergic diseases that are on the rise in industrialized countries [17]. Pharmacological targeting of the underlying pathological dysregulation of immune cells is the basis for a wide range of therapeutic applications [18,19]. The major components of the adaptive immune system are T and B cells [20]. Activation of peripheral T cells is a result of the engagement of both the T-cell receptor–CD3 complex and the CD28 costimulatory receptor leading to T-cell proliferation and the production of IL-2 [21]. Similarly, B-cell activation is induced by a combination of the binding of ligand to the CD40 receptor and co-stimulation with interleukins such as IL-4 and IL-21 [22,23]. Signal transduction from the stimulated IL-2, IL-4, or IL-21 receptors is initiated by the activation of receptor-associated kinases from the Janus kinase (JAK) family (JAK-1, JAK-3, and Tyrosine kinase 2) and downstream activation (phosphorylation) of the STAT family [24,25]. This occurs via signal transduction pathways with the subsequent phosphorylation and translocation of dimerized STAT5 proteins, activated MAPK, or p70 S6 kinase to the nucleus, stimulating transcription [26]. In consequence, the STAT intracellular signaling pathways provide multiple targets for the specific modulation of lymphocyte activity. Next, an increased surface expression of receptors such as CD69 and CD25 (IL-2RA alpha chain) is characteristic of activation of both T- and B-cells activation [27,28,29]. Similarly, human lymphocyte antigen isotype DR (HLA-DR), a human class II major histocompatibility complex antigen, is expressed on B cells and appears during later stages of activation on activated T cells and natural killer lymphocytes [29]. In response to lymphocyte activation, cytotoxic CD8+ T cells undergo a characteristic kinetic sequence upon activation that includes rapid proliferation and expansion followed by population contraction due to cell death and differentiation into various effector cell subtypes [30]. CD4+ T cells function as both helper and regulatory cells that are not only necessary for the proper function of immune response but also contribute to different pathological processes [31]. The basic function of B cells is the production of immunoglobulins, which is tightly regulated under normal (healthy) conditions at both the molecular and cellular level [32]. 

According to previous studies, pseurotins possess a wide range of biological activities including their effects on the immune system. However, the effects of pseurotins on the functional response of human lymphocytes are still unknown. Therefore, a complex characterization of the effects of pseurotin D on human lymphocytes was performed by determining T-cell and B-cell proliferation and activation together with the activation of the STAT signaling pathway.

## 2. Results

### 2.1. Pseurotin Inhibits the Activation of Both CD4+ and CD8+ T Cells

**Figure 1:**
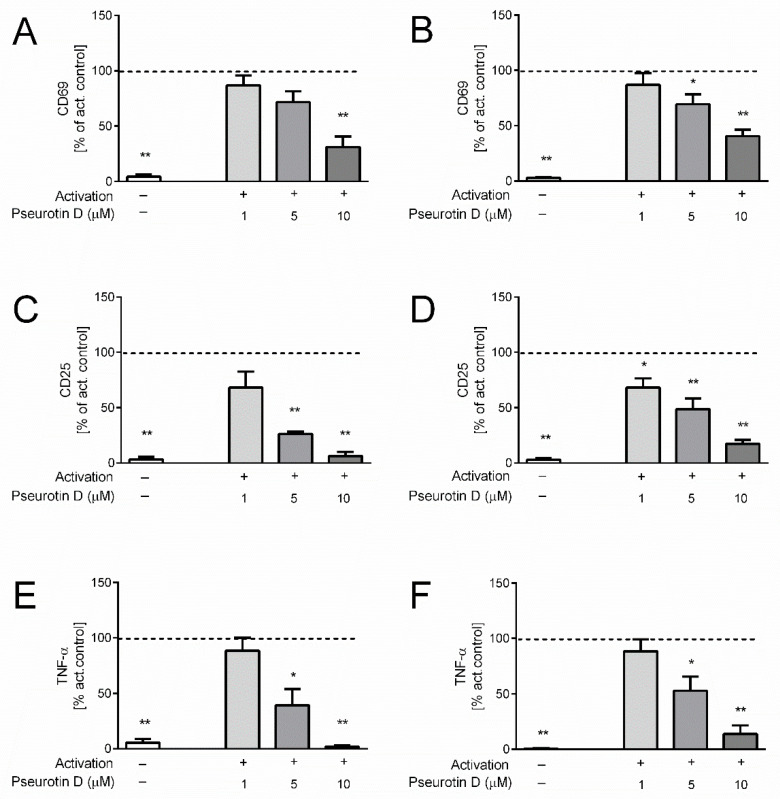
The effect of natural pseurotin D on the activation of human T cells. T cells were pretreated with pseurotin D (1–10 μM) for 30 min, then activated by anti-CD3 (1 μg/mL) and anti-CD28 (0.01 μg/mL). The expressions of activation markers were measured by flow cytometry after a 5-day incubation period. (**A**) The expression of marker CD69 on CD4+ T cells. (**B**) The expression of marker CD69 on CD8+ T cells. (**C**) The expression of marker CD25 on CD4+ T cells. (**D**) The expression of marker CD25 on CD8+ T cells. The production of proinflammatory cytokine TNF-α. (**E**) The production of TNF-α by CD4+ T cells. (**F**) The production of TNF-α by CD8+ T cells. Results are expressed as percentage of the activated control (dashed line indicates 100% of control). Data are expressed as the mean ± SEM. One sample t-test was used to analyze the significance of the obtained data by separately comparing the effect of each experimental treatment with an activated control (* *p* < 0.05, ** *p* < 0.01), *n* = 4. TNF: tumor necrosis factor, SEM: standard error of the mean.

Isolated human helper CD4+ and cytotoxic CD8+ cells were activated by a combination of antibodies against CD3 and CD28 for 5 days, which induced robust upregulation of the surface expressions of activation markers CD69 (Figure 1A,B) and CD25 (Figure 1C,D). Interestingly, the treatment of both CD4+ and CD8+ cells with pseurotin D dose-dependently inhibited their activation (Figure 1). In the case of CD4+ cells, pseurotin D significantly decreased the expression of CD69 at a concentration of 10 μM and the expression of CD25 at concentrations of 5 and 10 µM compared to the untreated activated control (Figure 1A,C). In the case of CD8+ cells, pseurotin D significantly decreased the expression of CD69 at concentrations of 5 and 10 µM and the expression of CD25 at all tested concentrations of 1, 5, and 10 µM compared to the untreated activated control (Figure 1B,D). The activation of the cells also stimulated the massive production of pro-inflammatory cytokine tumor necrosis factor (TNF)-α by human T cells (Figure 1E,F). Pseurotin D significantly inhibited the production of TNF-α by both CD4+ and CD8+ cells at concentrations of 5 and 10 µM.

### 2.2. Pseurotin Inhibits the Activation of STAT Signaling Pathways in T cells 

**Figure 2:**
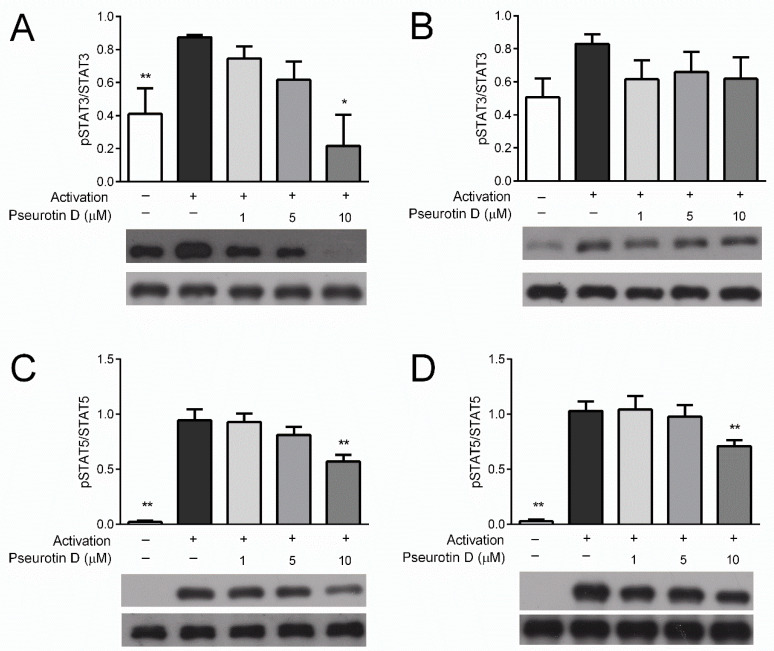
The effect of pseurotin D on the phosphorylation of STAT proteins in human T cells. T cells were pretreated with pseurotin D (1–10 μM) for 15 min, then activated by IL-2 (10 ng/mL). The phosphorylation of STAT3 (Tyr705) and STAT5 (Tyr694) was detected by Western blot after 30 min of incubation. (**A**) Phosphorylation of STAT3 in CD4+ T cells; (**B**) phosphorylation of STAT3 in CD8+ T cells; (**C**) phosphorylation of STAT5 in CD4+ T cells; (**D**) phosphorylation of STAT5 in CD8+ Table 0. ** *p* < 0.01), *n* = 3. STAT: signal transducers and activators of transcription, IL: interleukin.

The STAT signaling pathway plays a key role in the activation of T cells. To understand whether pseurotin D affects this pathway, STAT phosphorylation analysis was performed. The analysis compared the amount of phosphorylated STATs relative to the total abundance of these signaling proteins. The phosphorylation of STAT3 (Tyr705) and STAT5 (Tyr694) in CD4+ T cells was inhibited dose-dependently with significance after treatment with pseurotin D at a concentration of 10 μM (Figure 2A,C). Similar trends were observed for CD8+ T cells (Figure 2B,D); however, a significant decrease occurred only in the case of STAT3 phospohorylation at a concentration of 10 μM. This is in close agreement with the inhibitory effects of pseurotin D on T-cell activation. 

### 2.3. Pseurotin Inhibits the Proliferation of T Cells

**Figure 3:**
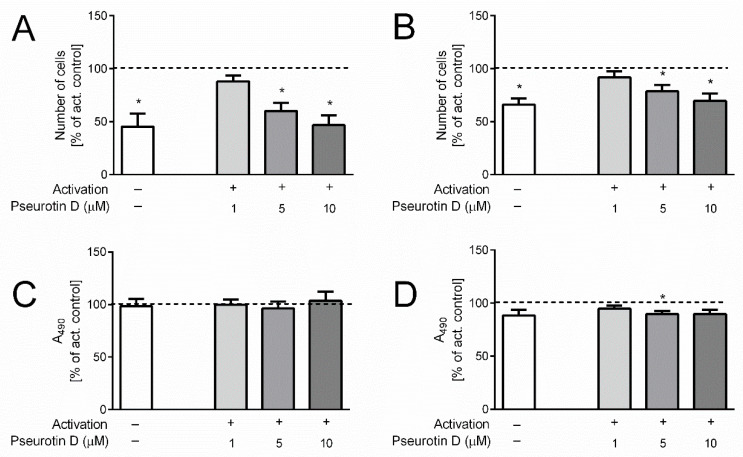
The effect of pseurotin D on the proliferation and viability of human T cells. T cells were pretreated with pseurotin D (1–10 μM) for 30 min, then activated by anti-CD3 (1 μg/mL) and anti-CD28 (0.01 μg/mL). The number of cells was measured by hemocytometer CASY after a 5-day incubation period. (**A**) The number of CD4+ T cells; (**B**) the number of CD8+ T cells. Acute toxicity was determined as the release of LDH into cell culture media. (**C**) LDH release by CD4+ T cells; (**D**) LDH release by CD8+ T cells. Results are expressed as a percentage of the control not treated by pseurotin D (dashed line indicates 100% of control). Data are expressed as the mean ± SEM. One sample *t*-test was used to analyze the significance of the obtained data by separately comparing the effect of each experimental treatment with an activated control (* *p* < 0.05), *n* = 3. LDH: lactate dehydrogenase.

The activation of the T cells was accompanied by their increased proliferation, as was determined by direct cell counting after 5 days incubation (368 ± 3 × 10^3^/mL control vs. 963 ± 20 × 10^3^/mL activated for CD4+ and 323 ± 15 × 10^3^/mL control vs. 488 ± 54 × 10^3^/mL activated for CD8+). The proliferation was inhibited after treatment with pseurotin D. In the case of CD4+ cells, the number of cells was significantly decreased at concentrations of 5 and 10 μM (Figure 3A). In the case of CD8+ cells, a significant reduction in the number of cells was observed in samples treated with 5 and 10 μM pseurotin D compared to untreated activated control (Figure 3B). 

**Figure 4:**
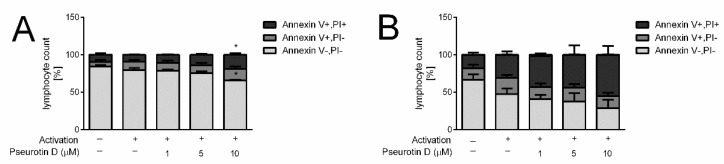
The effect of pseurotin D on the viability of human CD8+ and CD4+ T cells. Cells were cultivated with pseurotin D (1–10 μM) and with or without activators anti-CD3 (1 μg/mL) and anti-CD28 (0.01 μg/mL). Apoptotic and dead cells were identified based on Annexin V and PI staining after a 5-day incubation period. (**A**) Viability activated CD4+ T cells; (**B**) viability of activated CD8+ T cells. Results are expressed as a percentage of each population of viable (Annexin V-/PI-), apoptotic (Annexin V+/PI-), late apoptotic/dead (Annexin V+/PI+) cells in a particular sample. Data are expressed as the mean ± SEM. One-way ANOVA was used to analyze the significance of the obtained data by separately comparing the effect of each experimental treatment with an activated control (* *p* < 0.05), *n* = 4. PI: propidium iodide.

Importantly, this decrease in activated CD4+ and CD8+ cell numbers mediated by pseurotin D was not connected with acute toxicity, since the release of LDH by pseurotin D-treated cells was not increased compared to untreated control after the 5-day treatment (Figure 3C,D). In contrast, LDH levels were even slightly decreased in the case of CD8+ (Figure 3D). To further evaluate the viability of cells after the 5-day treatment, the surface expression of Annexin V together with cell membrane permeability was determined. As shown in Figure 4, the highest concentration of pseurotin D (10 μM) induced a significant decrease in the number of viable cells that had a non-permeable cell membrane and were positive for Annexin V staining.

### 2.4. Pseurotin D Affects the Differentiation of B Cells

**Figure 5:**
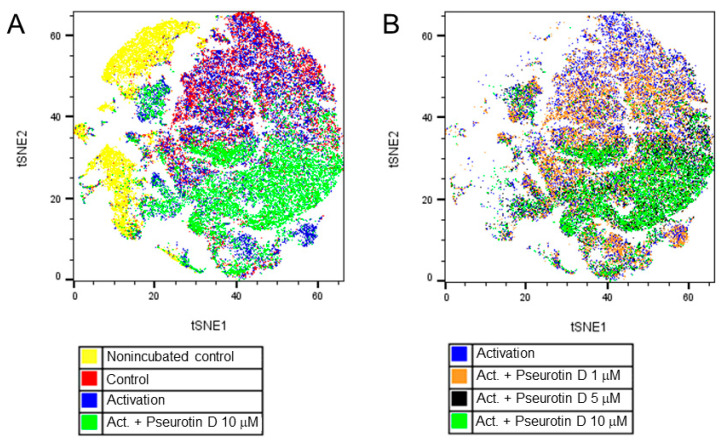
The effect of natural pseurotin D on differentiation of B cells. B cells were pretreated with pseurotin D (1–10 μM) for 30 min, then activated by a combination of IL-21 (50 ng/mL) and anti CD40 (1 μg/mL). The expression of surface markers was measured by flow cytometry after a 7-day incubation period. Data were analyzed by the tSNE algorithm. (**A**) Representative map of B-cell population after a 7-day incubation period; (**B**) Representative map of B-cell population after 7 days of incubation with pseurotin D (1–10 μM). tSNE: t-distributed stochastic neighbor embedding.

**Figure 6:**
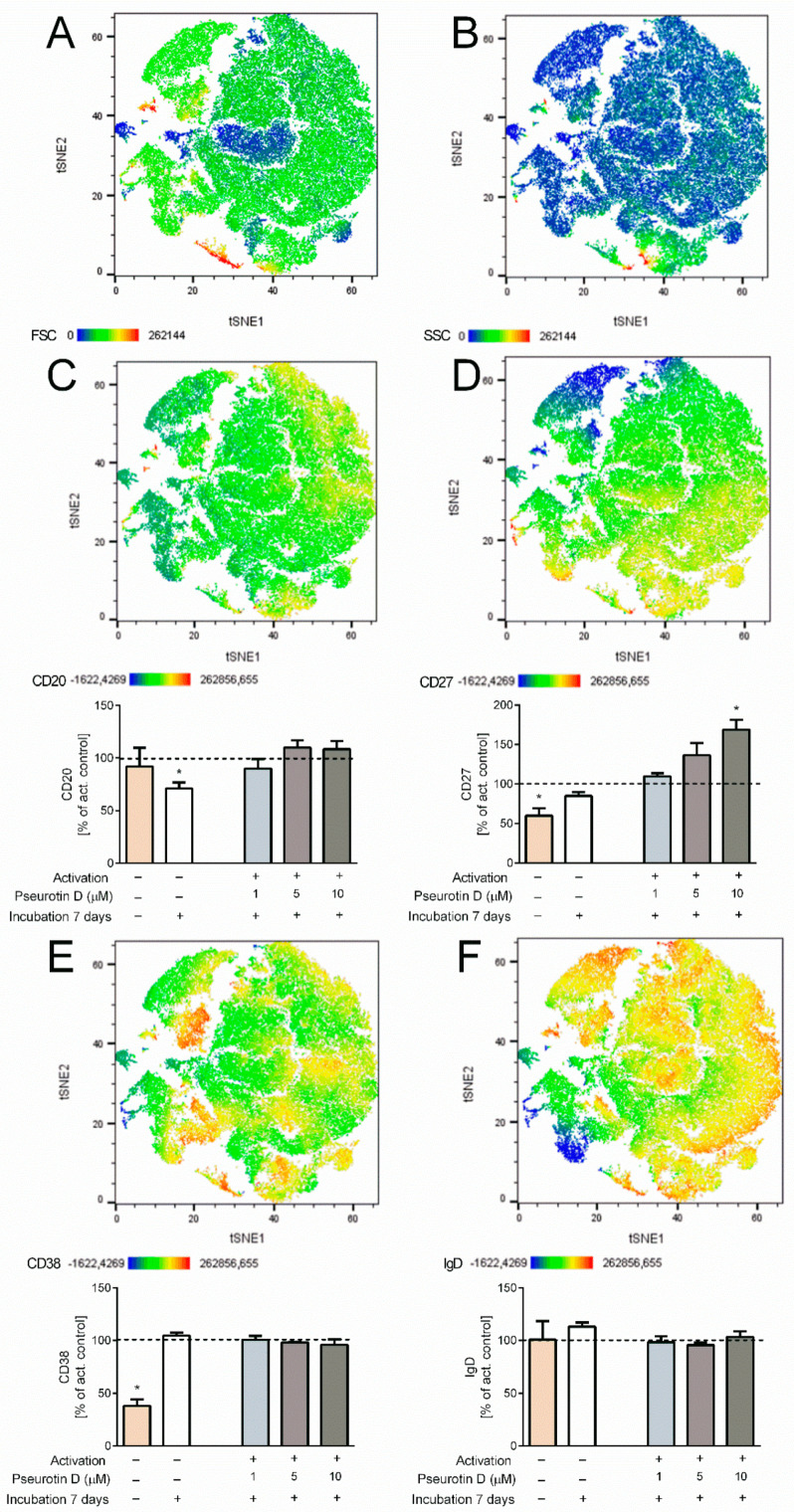
The effect of natural pseurotin D on differentiation markers on human B cells. B cells were pretreated with pseurotin D (1–10 μM) for 30 min, then activated by a combination of IL-21 (50 ng/mL) and anti-CD40 (1 μg/mL). The expressions of markers were measured by flow cytometry after a 7-day incubation period. (**A**) The size of cells—FSC detector; (**B**) the granularity of cells—SSC detector; (**C**) the expression of marker CD20 on B cells; (**D**) the expression of marker CD27 on B cells; (**E**) the expression of marker CD38 on B cells; (**F**) the expression of marker IgD on B cells. Data were analyzed by the tSNE algorithm. Results are expressed as a percentage of the activated control, expressed as the mean ± SEM and shown in bar graphs. One sample t-test was used to analyze the significance of the obtained data by separately comparing the effect of each experimental treatment with an activated control (* *p* < 0.05) *n* = 4. Ig: immunoglobulin.

In the case of B cells, changes in the surface expression of markers CD20, CD27, CD38, and IgD were determined to evaluate the effects of pseurotin D on B-cell differentiation. Interestingly, pseurotin D affected the differentiation of B cells, as visualized by the tSNE algorithm (Figure 5). The map of the population distribution is divided into two parts for better legibility. Figure 6 shows the effect of pseurotin D on particular parameters. The most affected marker was CD27, whose expression was increased dose-dependently with significance after treatment with pseurotin D at a concentration of 10 μM (Figure 6D). 

## 3. Discussion

In this study, we observed the inhibition of the anti-CD3/CD28-mediated activation of both CD4+ and CD8+ T cells by pseurotin D treatment in a dose-dependent manner, presented as the decreased proliferation and downregulated expression of activation markers (CD69 and CD25) and the production of TNF-α, which correspond with the inhibited phosphorylation of STAT3 and STAT5. In human B cells, pseurotin D did not significantly inhibit their activation; however, it affected their differentiation as it led to a complex change in the surface expressions of differentiation markers CD20, CD27, CD38, and IgD. This advances the current mechanistic understanding of the pseurotin-mediated modulation of the immune response. 

Our results support the assumption that pseurotin D can significantly inhibit the response of immune cells of both lymphoid or myeloid origin to selected activators. Previously, pseurotin D inhibited the endotoxin-mediated activation of RAW 264.7 murine macrophages in vitro, as was documented by the inhibition of IL-6 and nitric oxide release [14]. Furthermore, inhibition of the production of pro-inflammatory mediators including TNF-α and IL-6 by pseurotin A was observed in the synovial fluid of rats with rheumatoid arthritis [33]. The inhibition of the activation of both CD4+ and CD8+ T cells by pseurotin D was accompanied by a dose-dependent decline in T-cell proliferation. This decrease in the number of T cells was not accompanied by acute toxicity, as LDH release was not increased by pseurotin D. On the other hand, pseurotin D at the highest tested concentration (10 μM) induced apoptosis in T cells. We can speculate that both the inhibition of proliferation and the induction of apoptotic processes are connected with the inhibition of STAT signaling pathways essential for T-cell proliferation and survival [34,35]. In agreement with this, pseurotin D (10 μM) significantly inhibited the phosphorylation of both STAT3 and STAT5 in CD4+ and CD8+ T cells. The inhibition of STAT signaling pathways was also observed in our previous studies employing murine macrophages or splenocytes [14,15] and was similarly connected with the inhibition of murine macrophage proliferation by pseurotins [14]. In contrast, Ishikawa et al. did not observe any inhibitory effect of pseurotin A on T-cell proliferation induced by phytohemagglutinin [16]. However, in their model, the pseurotin analog synerazol inhibited T-cell proliferation. Meanwhile, Asami et al. observed that the natural pseurotin analog azaspirene inhibited angiogenesis [12], which was accompanied by decreased activation of the Raf-1 pathway [10]. Further, the inhibition of extracellular signal-regulated kinases (ERK) and p38 kinase and the activation of nuclear factor kappa B (NF-κB) transcription factor by pseurotin A was observed in cells from synovial tissue of rats with rheumatoid arthritis [33]. In bone marrow macrophages activated by the receptor activator of NF-κB ligand, pseurotin A reduced the phosphorylation of MAPK family members—namely, ERK, p-38, and c-Jun N-terminal kinases, as well as the activation of the NF-κB pathway [36]. Differences between the observations of various authors could be explained by the complex interconnectedness of signaling pathways activated by different receptors, which is further dependent on the particular type of immune cell. 

Antigen-driven B-cell proliferation, differentiation, affinity maturation, and immunoglobulin isotype switching depend on co-stimulatory signals, such as the stimulation of cytokine receptors IL-4 or IL-21 and the interaction between CD40 and CD40L [37]. In the case of activated human B cells, pseurotin D modulated the differentiation of B cells. The effects were visualized using a multiparametric-approach tSNE algorithm that included the surface expressions of CD20 (a marker of early phases of B-cell development), CD27 (a marker of memory B cells), CD38 (a marker of plasmablasts), and IgD (a marker of unswitched memory cells). Treatment with pseurotin D markedly changed cell distribution in the 2D tSNE graph. The decomposition of data into single parameters shows the expression of CD27 as being most affected by pseurotin D, the latter mediating an increase in CD27 expression. We also attempted to determine the effects of pseurotin D on human B-cell activation and proliferation in vitro. However, the IL-4-mediated activation of isolated B cells from the blood of healthy human donors induced only a very limited increase in the activation markers CD69, CD25, and HLA-DR—up to 20% above the values of the non-activated control (data not shown). Similarly, treatment for 7 days with the activators IL-4 or a combination of IL-21 and antibody against CD40 did not significantly affect the proliferation of B cells (332 ± 9 × 10^3^/mL control vs. 373 ± 9 × 10^3^/mL activated by IL-4; 355 ± 22 × 10^3^/mL control vs. 368 ± 32 × 10^3^/mL activated by the combination CD40L and IL-21). Thus, this experimental in vitro setup did not allow the effect of pseurotin on B-cell activation to be tested. However, in our previous study employing murine splenocytes, we observed the inhibition of the production of IgE, the inhibition of the proliferation of B cells, and the inhibition of STAT phosphorylation by pseurotin D [15]. Similarly, Ishikawa et al. observed that pseurotin A and several pseurotin analogs, including synerazol, significantly inhibited the production of IgE by isolated mouse splenocytes [16]. In sum, pseurotins have the potential to modulate B-cell response to physiologically relevant activators.

Overall, our data support previously reported immunomodulatory effects of pseurotins, which suggests a potential therapeutic usage of pseurotins in the treatment of diseases characterized by defective immune system such as allergic diseases, where especially CD4+ T cells play an important role. Moreover, natural pseurotins and their synthetic analogs were demonstrated to inhibit the production of IgE by murine splenocytes [15,16]. Further, a reduction of delayed-type hypersensitivity response by pseurotin D was observed using a murine in vivo model [15]. The therapeutic potential of pseurotin A was discussed in osteoclast-related bone diseases such as osteoporosis [36] and rheumatoid arthritis [33] during which loss of T cell tolerance occurs. Pseurotin A was also reported to suppress the progression of hormone-dependent BT-474 breast tumors in a nude mouse xenograft model [38]. Therefore, pseurotins could represent a potent new group of drugs for therapeutic treatment of pathological responses of the immune system.

## 4. Materials and Methods

### 4.1. Materials

Pseurotin D (cat # BVT-0426) was purchased from Adipogen Life Science (USA). A stock solution was prepared in dimethyl sulfoxide (DMSO) at a 10 mM concentration, and the cultivation medium was used for dilution to working concentrations. DMSO was not toxic to lymphocytes in the concentrations used, as proven by lactate dehydrogenase (LDH) tests (data not shown). Human recombinant IL-2 (cat # 200-02), IL-4 (cat # 200-04) and IL-21 (cat # 200-21) were purchased from PreproTech (USA). Anti-human CD3 (cat # 11-202), anti-human CD28 (cat # 11-577) and anti-human CD40L (cat # 10-416) were purchased from Exbio (Prague, Czech Republic). Stock solutions were prepared according to the manufacturer’s instructions.

### 4.2. Cell Isolation

Peripheral blood mononuclear cells (PBMCs) were isolated from the blood of healthy volunteers using dextran separation followed by Histopaque 1077 separation and lysis of the remaining erythrocytes by water hypotonic lysis. Blood samples were collected from healthy volunteers who had given their informed consent; the volunteers were aged 18–60, both female and male; they did not take any drugs and were free of any signs of cold or other diseases for at least 3 weeks before blood draw; the collection protocol was approved by The Ethics Committee for Research at Masaryk University, Brno, Czech Republic (number EKV-2018-083) approved in 5 November 2018. Monocytes were removed after their adherence to a plastic dish for 30 min. The remaining lymphocytes were sorted by BD FACS ARIA II (BD Biosciences, Franklin Lakes, NJ, USA) according to the specific surface markers. T-helper cells (CD4+) were stained using conjugate antibody anti-CD4-Pacific Blue (cat # 187120), T-cytotoxic cells (CD8+) were stained using conjugate antibody anti-CD8-FITC (cat # 105250), and B cells (CD19+) were stained using conjugate antibody anti-CD19-APC (cat # 2111060), all from Sony biotechnology (San Jose, CA, USA).

### 4.3. Activation and Treatment

Twenty-four-well cell culture plates were pre-treated with 0.3 mL PBS including 1 μg/mL anti-CD3 antibody (ExBio, Prague, Czech Republic) and incubated at 4 °C overnight. T cells were pretreated with pseurotin D (1, 5, 10 μM) for 30 min and seeded at a density of 3−4 × 10^5^ cells per mL in Roswell Park Memorial Institute medium (RPMI) 1640 containing 10% (*v/v*) low endotoxin fetal bovine serum (FBS LE) (Serana, Pessin, Germany) and 1% penicillin–streptomycin (Gibco, Dublin, Ireland). Then, soluble anti-CD28 antibody at a concentration of 0.01 μg/mL was added for co-activation. The cells were incubated for 5 days.

B cells were pretreated with pseurotin D (1, 5, 10 μM) for 30 min and seeded at a density of 3−4 × 10^5^ cells per mL in RPMI 1640 containing 10% (*v/v*) FBS LE and 1% penicillin–streptomycin. The cells were activated by IL-4 at a concentration of 15 ng/mL or by a combination of anti-CD40 antibody at a concentration of 1 μg/mL and IL-21 at a concentration of 50 ng/mL. The cells were incubated for 7 days.

For the purpose of detecting phosphorylation, T cells were seeded at a density of 3−4 × 10^5^ cells per mL in RPMI 1640 containing 10% (*v/v*) FBS LE and pretreated with pseurotin D (1, 5, 10 μM) for 15 min. Then, the cells were activated by IL-2 for 30 min. Background phosophorylation of STAT proteins was caused by FBS supplementation which also protected the cells against apoptosis (Supplementary Figures S1 and S2).

### 4.4. Cell Proliferation and Cytotoxicity Determination

Supernatants were collected after the tested incubation period (5 or 7 days) and numbers of cells were determined by a CASY cell counter (OLS-OMNI Life Science, Bremen, Germany). Cell lysis accompanying acute cell death (necrosis) was quantified by measuring the activity of LDH released from the cytosol of dying cells using a colorimetric assay employing Cytotoxicity Detection KitPLUS (Roche, Mannheim, Germany), as described previously [39]. Each time, a positive control was prepared by reflecting the maximum LDH released by cells after the application of lysis buffer (a part of the kit) for 30 min. The supernatant was then used for the colorimetric reaction. The absorbance was measured at 490 nm using a Sunrise microplate reader (Tecan, Männedorf, Switzerland). 

### 4.5. Determination of Activation Markers

The surface expressions of CD69 (anti-CD69-APC/Cy7, # 2154570) and CD25 (anti-CD25-PE/Cy7 # 2113060) were used as markers of T cell activation status. In the case of B cells, the surface expressions of CD69, CD25, and HLA-DR (anti-HLA-DR-PE/Cy5 # 2138040) were used as markers of activation status. The surface expressions of CD20 (anti-CD20-FITC # 2111515), CD27 (anti-CD27-BV421 # 2114115), CD38 (anti-CD38-APC/Cy7 # 2117665), and IgD (anti-IgD- PerCP/Cy5.5 # 2341035) were used as markers of the differentiation of B cells. All antibodies were purchased from Sony Biotechnology (San Jose, CA, USA). The cells were centrifuged, resuspended in PBS containing 0.1% bovine serum albumin (BSA) (Sigma, St. Louis, MO, USA), and incubated with fluorescently labeled antibodies on ice for 30 min for surface staining. Then, the cells were washed with PBS and analyzed by a BD FACSVerse flow cytometr (BD Biosciences, Franklin Lakes, NJ, USA). The data were evaluated in FlowJo software (Becton, Dickinson & Company, Franklin Lakes, NJ, USA) and expressed as the geometric mean of fluorescence. The effect on the differentiation of B cells was evaluated by a t-distributed stochastic neighbor embedding (tSNE) algorithm considering FSC, SSC, CD20, CD27, CD38, and IgD. Analysis by tSNE algorithm was performed in FlowJo software. Parameters were set as follows: iterations, 5000; perplexity, 100; learning rate, 4200; Learning configuration, Auto (opt-tSNE); KNN algorithm, Exact; gradient algorithm, Barnes-Hut.

### 4.6. Apoptosis Detection

Apoptotic and dead cells were determined on the basis of staining with Annexin V conjugated with FITC (Exbio, Prague, Czech Republic) and propidium iodide (PI). The cells were resuspended in Annexin V binding buffer containing Annexin V conjugated with FITC (1:150). PI was added just before analysis, which was performed by BD FACSVerse flow cytometr.

### 4.7. Production of Cytokines 

The production of TNF-α was detected in culture media after 5 days of incubation by TNF-α Human Uncoated ELISA Kit (Thermo Fisher Scientific, Waltham, MA, USA), as previously described by [40], employing a Sunrise microplate reader. 

### 4.8. Western Blot

Cells were lysed using lysis buffer (1% sodium dodecyl sulfate (SDS); 100 mM Tris, pH 7.4; 10% glycerol) containing phosphatase and protease inhibitor cocktail tablets (Roche, Mannheim, Germany). The protein concentration was determined using BCA™ protein assay (Pierce, Rockford, IL, USA) with BSA used as a standard. The expressions of target proteins were quantified by Western blot analysis, as described previously [14]. The same amount of proteins (30 μg) from each sample was supplemented with Laemli buffer (200 mM Tris-HCl, pH 6.8; 3% SDS; 30% glycerol; 0.03 bromphenol blue; 3% ß-mercaptoethanol; 200mM dithiothreitol) and subjected to SDS-polyacrylamide gel electrophoresis, using 10% running gel. Separated proteins were transferred to polyvinylidene difluoride membranes that were blocked in 5% non-fat milk in Tris-buffered saline with 0.1% Tween 20 (TBS-T). Then, the membranes were incubated with antibodies against β-actin (1:5000, sc-47778, Santa Cruz Biotechnology, Dallas, TX, USA), STAT3 (1:1000; # 12640, Cell signaling technologies, Danvers, MA, USA), p-STAT3 (1:300; #9145, Cell signaling technologies, Danvers, MA, USA), STAT5 (1:1000; #9363, Cell signaling technologies, Danvers, MA, USA), and p-STAT5 (1:1000; #4322, Cell signaling technologies, Danvers, MA, USA) in 5% non-fat milk/TBS-T at 4 °C overnight. Corresponding secondary HRP-conjugated anti-rabbit or anti-murine antibodies (1:3000 or 1:2000 in 5% non-fat milk/TBS-T, 1h, at room temperature; Sigma, St. Louis, MO, USA) were employed. The immunoreactive bands were detected using an ECL detection reagent kit (Pierce, Rockford, IL, USA) and exposed to radiographic film (AGFA, Mortsel, Belgium). Optical densities were quantified by scanning densitometry and expressed in arbitrary units determined by ImageJ software, v1.52n (NIH, Bethesda, MD, USA).

### 4.9. Statistical Analysis

Data are presented as mean ± standard error of the mean (SEM). Statistical analysis was performed using Prism-6.01 software (GraphPad Software, La Jolla, CA, USA). Statistical differences were tested by one-way ANOVA or one-sample t-test to compare values expressed as percentages. In the case of one sample t-test application, the Bonferroni correction of the *p*-value for multiple comparisons was performed. The number of independent repeats (n) is given in each figure legend.

## 5. Conclusions

On the basis of our data, we suggest that pseurotin D has the potential to inhibit T-cell activation, including T-cell proliferation, the expression of surface markers, and the production of pro-inflammatory mediators. Concerning B cells, pseurotin D did not significantly inhibit their activation; however, it affected their differentiation as it led to a complex change in the surface expression of differentiation markers. Overall, these data widen our knowledge of the pseurotin-mediated modulation of immune response and support the vision that the unique structure of pseurotins can provide inspiration for the design of new chemotypes of small molecules targeting pathologically defective immune system functions under disease conditions.

## Data Availability

The data presented in this study are available on request from the corresponding author.

## Supplementary Materials

The following are available online at https://www.mdpi.com/1422-0067/22/4/1938/s1, Figure S1: The effect of FBS on the viability of human CD4+ T cells. Figure S2: The effect of FBS on the phosphorylation of STAT proteins in human CD4+.

## References

[1] Varga J., Frisvad J.C., Samson R.A. (2011). Two new aflatoxin producing species, and an overview of *Aspergillus* section Flavi. Stud. Mycol..

[2] Maiya S., Grundmann A., Li X., Li S.M., Turner G. (2007). Identification of a hybrid PKS/NRPS required for pseurotin A biosynthesis in the human pathogen *Aspergillus fumigatus*. Chembiochem.

[3] Tsunematsu Y., Fukutomi M., Saruwatari T., Noguchi H., Hotta K., Tang Y., Watanabe K. (2014). Elucidation of pseurotin biosynthetic pathway points to trans-acting C-methyltransferase: Generation of chemical diversity. Angew. Chem. Int. Ed. Engl..

[4] Ishikawa M., Ninomiya T. (2008). Chemical modification of pseurotin A: One-pot synthesis of synerazol and pseurotin E and determination of absolute stereochemistry of pseurotin E. J. Antibiot..

[5] Bloch P., Tamm C., Bollinger P., Petcher T.J., Weber H.P. (1976). Pseurotin, a new metabolite of *Pseudeurotium ovalis* Stolk having an unusual hetero-spirocyclic system. Helv. Chim. Acta..

[6] Jo D., Han S. (2018). Total syntheses of spirocyclic PKS-NRPS-based fungal metabolites. Chem. Commun..

[7] Fisch K.M. (2013). Biosynthesis of natural products by microbial iterative hybrid PKS-NRPS. RSC Adv..

[8] Ando O., Satake H., Nakajima M., Sato A., Nakamura T., Kinoshita T., Furuya K., Haneishi T. (1991). Synerazol, a New Antifungal Antibiotic. J. Antibiot..

[9] Komagata D., Fujita S., Yamashita N., Saito S., Morino T. (1996). Novel neuritogenic activities of pseurotin A and penicillic acid. J. Antibiot..

[10] Asami Y., Kakeya H., Komi Y., Kojima S., Nishikawa K., Beebe K., Neckers L., Osada H. (2008). Azaspirene, a fungal product, inhibits angiogenesis by blocking Raf-1 activation. Cancer Sci..

[11] Igarashi Y., Yabuta Y., Sekine A., Fujii K., Harada K., Oikawa T., Sato M., Furumai T., Oki T. (2004). Directed biosynthesis of fluorinated pseurotin A, synerazol and gliotoxin. J. Antibiot..

[12] Asami Y., Kakeya H., Onose R., Yoshida A., Matsuzaki H., Osada H. (2002). Azaspirene: A novel angiogenesis inhibitor containing a 1-oxa-7-azaspiro[4.4]non-2-ene-4,6-dione skeleton produced by the fungus *Neosartotya* sp.. Organ. Lett..

[13] Anjum K., Bi H., Chai W., Lian X.Y., Zhang Z. (2017). Antiglioma pseurotin A from marine *Bacillus* sp. FS8D regulating tumour metabolic enzymes. Nat. Prod. Res..

[14] Vasicek O., Rubanova D., Chytkova B., Kubala L. (2020). Natural pseurotins inhibit proliferation and inflammatory responses through the inactivation of STAT signaling pathways in macrophages. Food Chem. Toxicol..

[15] Vasicek O., Fedr R., Skoroplyas S., Chalupa D., Sklenar M., Tharra P.R., Svenda J., Kubala L. (2020). Natural pseurotins and analogs thereof inhibit activation of B-cells and differentiation into the plasma cells. Phytomedicine.

[16] Ishikawa M., Ninomiya T., Akabane H., Kushida N., Tsujiuchi G., Ohyama M., Gomi S., Shito K., Murata T. (2009). Pseurotin A and its analogues as inhibitors of immunoglobulin E [correction of immunoglobuline E] production. Bioorg. Med. Chem. Lett..

[17] Gould H.J., Sutton B.J. (2008). IgE in allergy and asthma today. Nat. Rev. Immunol..

[18] Bluml S., McKeever K., Ettinger R., Smolen J., Herbst R. (2013). B-cell targeted therapeutics in clinical development. Arthritis Res. Ther..

[19] U.S. Department of Health and Human Services Centers for Disease Control and Prevention (2017). Summary Health Statistics for U.S. Adults: National Health Interview Survey.

[20] Rabb H. (2002). The T cell as a bridge between innate and adaptive immune systems: Implications for the kidney. Kidney Int..

[21] Li L., Yee C., Beavo J.A. (1999). CD3- and CD28-dependent induction of PDE7 required for T cell activation. Science.

[22] Hasbold J., Hong J.S., Kehry M.R., Hodgkin P.D. (1999). Integrating signals from IFN-gamma and IL-4 by B cells: Positive and negative effects on CD40 ligand-induced proliferation, survival, and division-linked isotype switching to IgG1, IgE, and IgG2a. J. Immunol..

[23] Avery D.T., Ma C.S., Bryant V.L., Santner-Nanan B., Nanan R., Wong M., Fulcher D.A., Cook M.C., Tangye S.G. (2008). STAT3 is required for IL-21-induced secretion of IgE from human naive B cells. Blood.

[24] Ding B.B., Yu J.J., Yu R.Y., Mendez L.M., Shaknovich R., Zhang Y., Cattoretti G., Ye B.H. (2008). Constitutively activated STAT3 promotes cell proliferation and survival in the activated B-cell subtype of diffuse large B-cell lymphomas. Blood.

[25] Ul-Haq Z., Naz S., Mesaik M.A. (2016). Interleukin-4 receptor signaling and its binding mechanism: A therapeutic insight from inhibitors tool box. Cytokine Growth Factor. Rev..

[26] Flynn M.J., Hartley J.A. (2017). The emerging role of anti-CD25 directed therapies as both immune modulators and targeted agents in cancer. Br. J. Haematol..

[27] Cibrian D., Sanchez-Madrid F. (2017). CD69: From activation marker to metabolic gatekeeper. Eur. J. Immunol..

[28] Kimura M.Y., Hayashizaki K., Tokoyoda K., Takamura S., Motohashi S., Nakayama T. (2017). Crucial role for CD69 in allergic inflammatory responses: CD69-Myl9 system in the pathogenesis of airway inflammation. Immunol. Rev..

[29] Reddy M., Eirikis E., Davis C., Davis H.M., Prabhakar U. (2004). Comparative analysis of lymphocyte activation marker expression and cytokine secretion profile in stimulated human peripheral blood mononuclear cell cultures: An in vitro model to monitor cellular immune function. J. Immunol. Methods.

[30] Marchingo J.M., Kan A., Sutherland R.M., Duffy K.R., Wellard C.J., Belz G.T., Lew A.M., Dowling M.R., Heinzel S., Hodgkin P.D. (2014). T cell signaling. Antigen affinity, costimulation, and cytokine inputs sum linearly to amplify T cell expansion. Science.

[31] Zhu J., Paul W.E. (2008). CD4 T cells: Fates, functions, and faults. Blood.

[32] Geha R.S., Jabara H.H., Brodeur S.R. (2003). The regulation of immunoglobulin E class-switch recombination. Nat. Rev. Immunol..

[33] Chen T., Zhang B., Lin Y., Pan J., Zhao X.X., Ren S.X. (2019). Protective Effects of Prunasin A against the Differentiation of Osteoclasts and Destruction of Cartilage via the Receptor Activator of Nuclear Factor-Kappa-Beta Ligand/Mitogen-Activated Protein Kinase/Osteoprotegerin Pathway in a Rat Model of Arthritis. Pharmacology.

[34] Moriggl R., Topham D.J., Teglund S., Sexl V., McKay C., Wang D., Hoffmeyer A., van Deursen J., Sangster M.Y., Bunting K.D. (1999). Stat5 is required for IL-2-induced cell cycle progression of peripheral T cells. Immunity.

[35] Rajasingh J., Raikwar H.P., Muthian G., Johnson C., Bright J.J. (2006). Curcumin induces growth-arrest and apoptosis in association with the inhibition of constitutively active JAK-STAT pathway in T cell leukemia. Biochem. Biophys. Res. Commun..

[36] Chen K., Qiu P.C., Yuan Y., Zheng L., He J.B., Wang C., Guo Q., Kenny J., Liu Q., Zhao J.M. (2019). Pseurotin A Inhibits Osteoclastogenesis and Prevents Ovariectomized-Induced Bone Loss by Suppressing Reactive Oxygen Species. Theranostics.

[37] Kienzler A.K., Rizzi M., Reith M., Nutt S.L., Eibel H. (2013). Inhibition of human B-cell development into plasmablasts by histone deacetylase inhibitor valproic acid. J. Allergy Clin. Immunol..

[38] Abdelwahed K.S., Siddique A., Mohyeldin M.M., Qusa M.H., Goda A.A., Singh S.S., Ayoub N.M., King J.A., Jois S.D., El Sayed K.A. (2020). Pseurotin A as a novel suppressor of hormone dependent breast cancer progression and recurrence by inhibiting PCSK9 secretion and interaction with LDL receptor. Pharmacol. Res..

[39] Georgiev Y.N., Paulsen B.S., Kiyohara H., Ciz M., Ognyanov M.H., Vasicek O., Rise F., Denev P.N., Lojek A., Batsalova T.G. (2017). Tilia tomentosa pectins exhibit dual mode of action on phagocytes as beta-glucuronic acid monomers are abundant in their rhamnogalacturonans I. Carbohydr. Polym..

[40] Vasicek O., Lojek A., Ciz M. (2020). Serotonin and its metabolites reduce oxidative stress in murine RAW264.7 macrophages and prevent inflammation. J. Physiol. Biochem..

