# Supplementary material for: Pseurotin D Inhibits the Activation of Human Lymphocytes
Source: Int J Mol Sci. 2021 Feb 16;22(4):1938. doi: 10.3390/ijms22041938 (PMC7920033; doi:10.3390/ijms22041938)
Supplement: Supplementary file 1 [file ijms-22-01938-s001.pdf]

SUPPLEMENTARY FIGURES FOR MANUSCRIPT:

Pseurotin D inhibits the activation of human lymphocytes

Daniela Rubanová[1,2], Petra Daďová[1,2], Ondřej Vašíček[1,2*], Lukáš Kubala[1,2,3 *]

[1] Institute of Biophysics of the Czech Academy of Sciences, 612 65, Brno, Czech Republic

[2] Institute of Experimental Biology, Faculty of Science, Masaryk University, Brno, 625 00, Czech Republic

[3]International Clinical Research Center, St. Anne's University Hospital, 656 91, Brno, Czech Republic

Corresponding authors:
Lukas Kubala, Ph.D.
Institute of Biophysics of the Czech Academy of Sciences
Kralovopolska 135, 612 65 Brno
E-mail: kubalal@ibp.cz
Phone: +420 – 541 517 117

Ondřej Vašíček, Ph.D.
Institute of Biophysics of the Czech Academy of Sciences
Kralovopolska 135, 612 65 Brno
E-mail: ondrej.vasicek@ibp.cz
Phone: +420 – 541 517 207

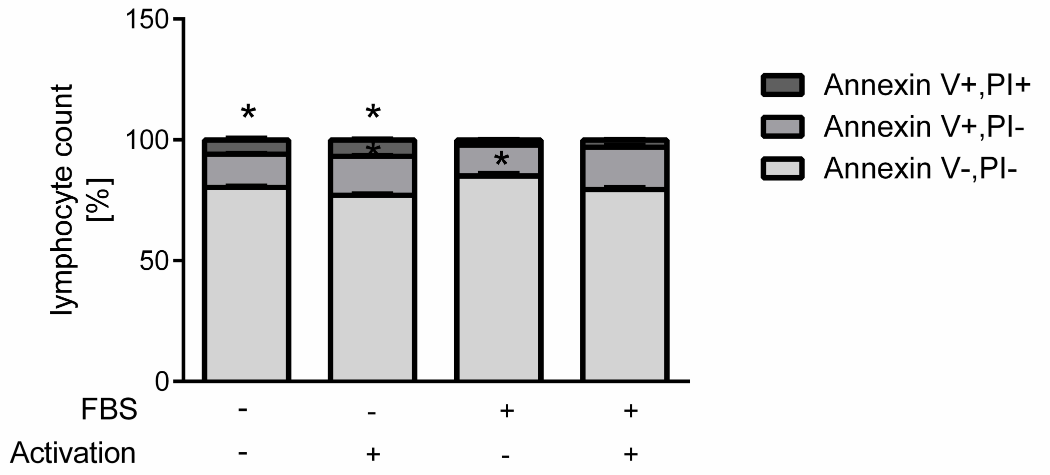

Supplementary figure 1: The effect of FBS on the viability of human CD4^{+} T-cells.

Cells were cultivated with or without 10% FBS and with or without activators anti-CD3 (1 μg/ml) and anti-CD28 (0.01 μg/ml). Apoptotic and dead cells were identified based on Annexin V and PI staining after a 2-day incubation period. Results are expressed as percentage of each population of viable (Annexin V^{-}/PI^{-}), apoptotic (Annexin V^{+}/PI^{-}), late apoptotic/dead (Annexin V^{+}/PI^{+}) cells in a particular sample. Data are expressed as the mean ± SEM. One-way ANOVA was used to analyze the significance of the obtained data by separately comparing the effect of each experimental treatment with an activated control (* $p < 0.05$), n = 3.

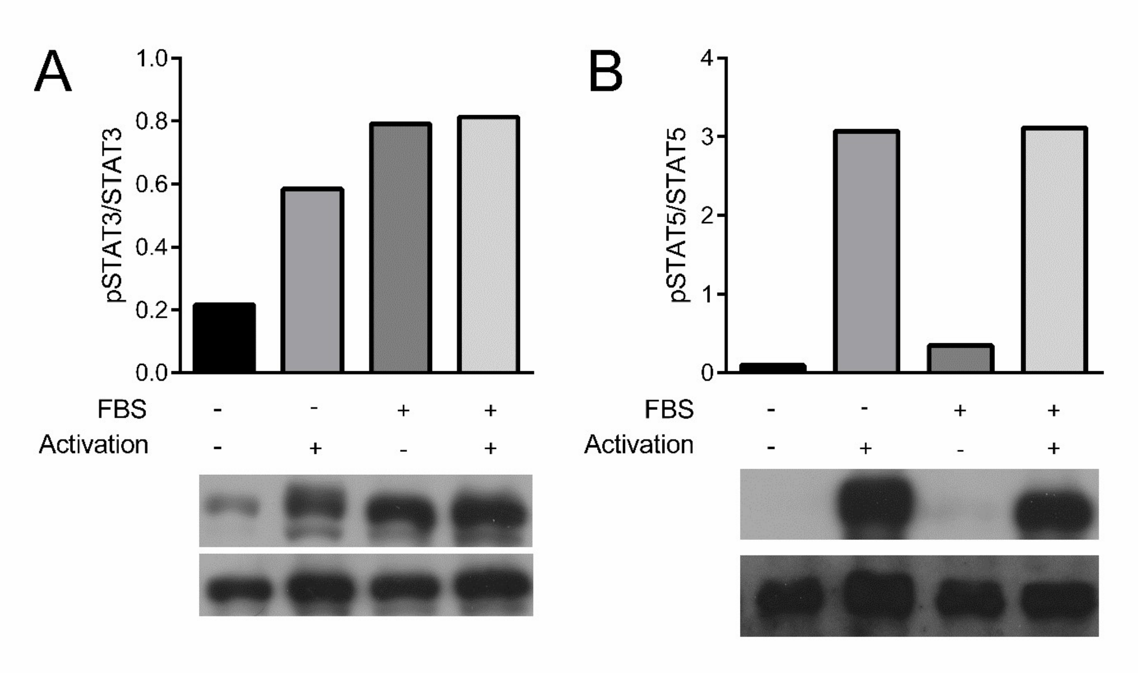

Supplementary figure 2: The effect of FBS on the phosphorylation of STAT proteins in human CD4⁺ T-cells. Cells were cultivated with or without 10% FBS and with or without activators anti-CD3 (1 μg/ml) and anti-CD28 (0.01 μg/ml). The phosphorylation of STAT3 (Tyr705) and STAT5 (Tyr694) was detected by western blot after 2 days of incubation. **A**) phosphorylation of STAT3 in $CD4^+$ T-cells; **B**) phosphorylation of STAT5 in $CD4^+$ T-cells. Representative western blots of the phosphorylated forms (pSTAT) and total forms (STAT) of proteins are shown below column plots. Results are expressed as the ratio of the optical density of the phosphorylated form to the optical density of total protein.
